# Cationic domains in particle-forming and assembly-deficient HBV core antigens capture mammalian RNA that stimulates Th1-biased antibody responses by DNA vaccination

**DOI:** 10.1038/s41598-018-32971-5

**Published:** 2018-10-02

**Authors:** Jana Krieger, Katja Stifter, Petra Riedl, Reinhold Schirmbeck

**Affiliations:** Department of Internal Medicine I, Ulm University Hospital, Ulm, Germany

## Abstract

The HBV core protein self-assembles into particles and encapsidates immune-stimulatory bacterial RNA through a cationic COOH-terminal (C150–183) domain. To investigate if different cationic domains have an impact on the endogenous RNA-binding of HBV-C antigens in mammalian cells, we developed a strep-tag (st) based expression/purification system for HBV-C/RNA antigens in vector-transfected HEK-293 cells. We showed that HBV-stC but not HBV-stC149 particles (lacking the cationic domain) capture low amounts of mammalian RNA. Prevention of specific phosphorylation in cationic domains, either by exchanging the serine residues S155, S162 and S170 with alanines (HBV-stCAAA) or by exchanging the entire cationic domain with a HIV-tat_48–57_-like sequence (HBV-stC149tat) enhanced the encapsidation of RNA into mutant core particles. Particle-bound mammalian RNA functioned as TLR-7 ligand and induced a Th1-biased humoral immunity in B6 but not in TLR-7^−/−^ mice by exogenous (protein) and endogenous (DNA) vaccines. Compared to core particles, binding of mammalian RNA to freely exposed cationic domains in assembly-deficient antigens was enhanced. However, RNA bound to non-particulate antigens unleash its Th1-stimulating adjuvant activity by DNA- but not protein-based vaccination. Mammalian RNAs targeted by an endogenously expressed antigen thus function as a natural adjuvant in the host that facilitates priming of Th1-biased immune responses by DNA-based immunization.

## Introduction

Plasmid DNA vaccination is an attractive technique to induce antigen-specific humoral and cellular immune responses^1,2^. Vector-encoded antigens are expressed in *in vivo* transfected APCs of the host. Antigens and/or antigenic material, released from non-professional antigen-expressing APCs (e.g., myocytes) to professional APCs (e.g. DCs) facilitated priming of immune responses^3^. Multiple laboratories have identified methods to optimize vector-driven transcription and protein expression, and/or to improve the immunogenicity of DNA-encoded antigens by co-delivering immune stimulating adjuvants, co-expressing immune modulatory cytokines or molecules stimulating the innate immune system^2^. In particular, the local induction of an inflammatory milieu at the side of vaccine injection was thought to attract professional APCs and thereby favour *de novo* priming of Th1-biased immune responses^4^.

The innate immune system has evolved endo/lysosomal and cytoplasmic pattern recognition receptors (PRRs) for the detection of pathogen-associated molecular patterns (PAMPs) like foreign nucleic acids or conserved molecules and structures of an invading pathogen^5^. Toll-like receptors (TLRs) can discriminate between different microbial nucleic acids, such as double-stranded (ds) RNAs (recognized by TLR-3), single-stranded (ss) RNAs (recognized by TLR-7 or TLR-8) or bacterial DNAs containing unmethylated CpG motifs (recognized by TLR-9). Nucleic acid-sensing TLRs are sequestered in an endo/lysosomal compartment and induce complex signaling cascades that result in the production of proinflammatory cytokines, chemokines and type I interferons^5^. These signals attract and activate professional APCs (DCs), which play a crucial role for priming adaptive immune responses^4,6^. Under certain conditions, mammalian self RNAs can also stimulate TLR-3- and/or TLR-7-mediated immune responses, for example Sm/RNP associated with lupus autoantigens^7–9^ or after release from damaged cells^10^. We thus hypothesized that cellular RNAs are potent “helper” molecules which, when captured by vector-expressed endogenous antigens may function as molecular adjuvant^11–13^.

An attractive antigen for targeting an endogenous, RNA-based adjuvant activity is the HBV core (HBV-C) protein. HBV-C self-assembles into particles that encapsidate nucleic acids, in particular pregenomic RNA (pgRNA) in HBV-infected cells or non-specific heterologous RNA in bacterial or yeast expression systems^14,15^ through a cationic COOH-terminal (C150–183) domain. Heterologous bacterial RNA bound to recombinant HBV-C particles specifically stimulates the innate immune system via the TLR-7 and induces a vigorous Th1-biased serum antibody response in mice^16,17^. Similarly, bacterial RNA encapsidated into recombinant bacteriophage virus-like particles displaying peptides of the human papillomavirus capsid protein L2 induces a Th1-biased serum antibody response in mice^18^. Particle-bound bacterial RNA has a >1000-fold higher potency as a Th1-inducing adjuvant than free RNA mixed to a protein antigen^16^. This suggested that encapsidation of heterologous RNA in particulate structures is a prerequisite for delivering RNA into APCs of the host. Furthermore, there is evidence that RNA bound to the cationic C150–183 domain of endogenously expressed HBV-C particles in mammalian cells exert a specific adjuvant activity: HBV-C, but not HBV-C149 and HBV-E antigens (lacking the cationic domain) bound [^3^H]-uracil-labelled cellular RNA in vector-transfected cells and stimulated a Th1-biased core-specific humoral immunity in mice by DNA vaccination with the gene gun^16^.

In this study, we investigated the non-specific binding of mammalian RNA to different cationic domains at the COOH terminus of HBV-C. We generated expression vectors that encode particle-forming and assembly-deficient HBV-C antigens and developed a strep-tag (st) based expression/purification system for HBV-C/RNA complexes in transiently transfected human HEK-293 cells. To elucidate the adjuvant activity of mammalian RNA captured by HBV-C antigens, we vaccinated B6 and TLR-deficient mice with recombinant antigens (exogenous antigens) or antigen-expressing vector DNA (endogenous antigens) and determined priming of Th1/Th2-biased core-specific antibody responses.

## Results

### HBV core antigens containing different cationic domains self-assemble into particles that capture mammalian RNA

**Figure 1:**
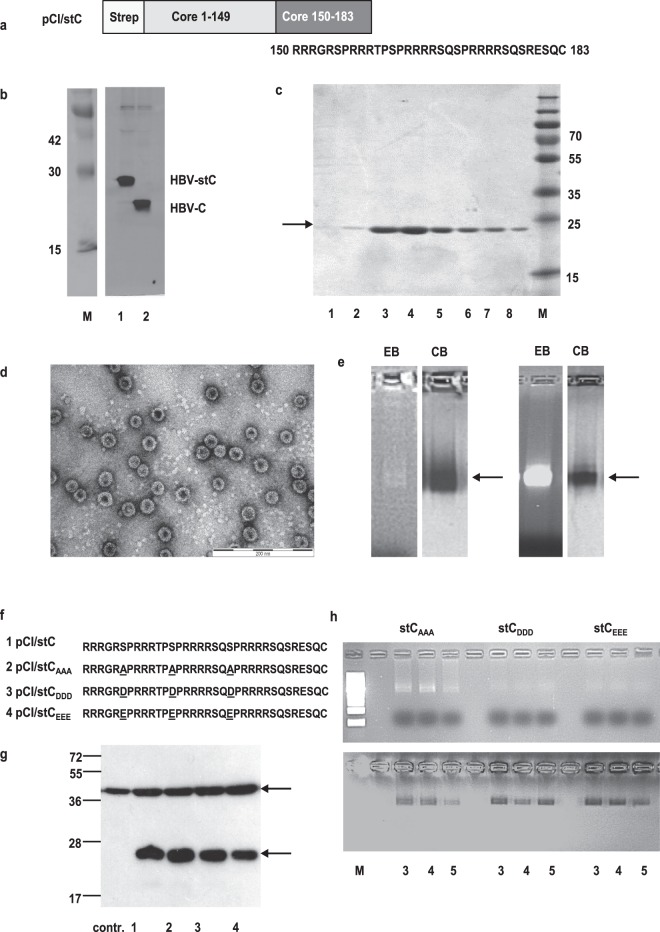
Expression and production of HBV core antigens. (**a**) Map of HBV-stC antigen that contains a NH2-terminal strep- tag sequence. The cationic C150-183 domain is shown. (**b**) HEK-293 cells were transiently transfected with pCI/stC (lane 1) or pCI/C (lane 2) and labelled with ^35^S-methionine/cysteine (left panel). Cell lysates were immunoprecipitated with a polyclonal rabbit anti HBV-C serum and processed for SDS-PAGE and fluorography of the gels. The original fluorographies of the gel used to generate this cropped figure are shown in Supplementary Fig. S1. (**c**) 5 × 10^8^ HEK-293 cells were transiently transfected with the pCI/stC vector and purified as described in the M&M section. 10 μl samples were processed for SDS-PAGE analysis followed by Coomassie Blue staining of the gel. Molecular weight marker (in kDa) is shown. Fractions with the highest antigen content (**c**; 3 and 4) were pooled, concentrated and processed for electron microscopy (the indicated scale bar represents 200 nm) (**d**) or for native agarose gel electrophoresis followed by ethidium bromide (EB) and subsequent Coomassie Blue (CB) staining of the gels (**e**; left panels). Furthermore, HBV-stC particles produced in bacteria were purified as described in Supplementary Information and analyzed on a separate agarose gel (**e**; right panels). The original gels used to generate this cropped figure are shown in Supplementary Fig. S3. (**f**) Schematic presentation of mutant cationic domains. The substitution of serine residues at positions S155, S162 and S170 in the wt HBV-stC to alanine HBV-stCAAA, aspartic acid HBV-stCDDD or glutamic acid HBV-stCEEE are indicated. (**g**) Lysates of HEK-293 cells transiently transfected with control pCI (contr.), pCI/stC (lane 1), pCI/stC_AAA_ (lane 2), pCI/stC_DDD_ (lane 3) or pCI/stC_EEE_ (lane 4) vectors were processed on SDS-PAGE followed by western blot analysis as described in Supplementary Information. The positions of beta-actin (upper panel) and respective HBV core antigens (lower panel) are indicated. (**h**) The respective antigens were produced in HEK-293 cells and fractions 3 to 5 were processed for native agarose gel electrophoresis followed by ethidium bromide (EB) and subsequent Coomassie Blue (CB)-staining of the gels.

The HBV-C protein contains a 34-residue cationic domain (C150–183) at its COOH-terminus (Fig. 1a). HBV-C and HBV-C149 (lacking the cationic domain) proteins self-assemble into particles, but only HBV-C particles non-specifically encapsidate RNA in bacterial expression systems^19^. We hypothesized that HBV-C may also bind mammalian RNA when selectively expressed in cells transfected with recombinant vector DNA or, relevant for DNA-based immunization, expressed in murine APCs targeted by plasmid DNA injection. To investigate particle formation and RNA-binding of different HBV core antigens under standardized conditions, we developed an expression system in human HEK-293 cells that allowed affinity purification of proteins under physiological conditions^20^. We used a HBV-C expressing pCI/C vector and cloned a streptavidin-binding tag (strep-tag or st) NH_2_-terminally to the HBV-C sequence. This generated the eukaryotic pCI/stC expression vector (Fig. 1a). Both, pCI/C and pCI/stC vectors expressed comparable levels of HBV-C and HBV-stC antigens in transiently transfected HEK-293 cells (Fig. 1b; Supplementary Figs S1 and S2), indicating non-disturbed expression of the HBV-stC protein by its NH_2_-terminal strep-tag modification.

For large-scale production, we transiently transfected 5–10 × 10^8^ HEK-293 cells with the pCI/stC vector. Cells were lysed 48 hours post transfection. The HBV-stC fusion protein was precipitated using a StrepTactin sepharose-packed column and eluted with desthiobiotin^20^. SDS-PAGE analysis of purified samples revealed a single HBV-stC protein band in the different fractions (Fig. 1c, lanes 1–8). The NH_2_-terminal strep-tag was thus accessible for recognition by the StrepTactin sepharose matrix and could be used to purify the HBV-stC protein (to >97% purity) from cell lysates. Electron microscopy of purified HBV-stC confirmed its particulate structure (Fig. 1d). Mammalian RNA (stained with ethidium bromide, EB) co-migrated with HBV-stC particles (stained with Coomassie Blue, CB) in native agarose gels (Fig. 1e, left panels; Supplementary Fig. S3a), demonstrating a tight interaction between particles and RNA. However, HBV-stC particles produced in HEK-293 cells contained lower amounts of heterologous RNA than HBV-stC particles expressed in bacteria (Fig. 1e, right panel; Supplementary Fig. S3b). The HBV-stC149 protein (lacking the cationic COOH-terminus) was efficiently expressed in transiently transfected HEK-293 cells but did not bind RNA (Supplementary Figs S2 and S4). Purified HBV-stC preparations almost quantitatively contained particles that did not migrate into naïve polyacrylamide gels under non-denaturing conditions (Supplementary Fig. S5). In contrast, HBV-stC149 preparations contained particles, but also a considerable proportion of non-particulate antigen migrating into the gels (Supplementary Fig. S5). As compared to HBV-stC particles, HBV-stC149 particle formation and/or stability was thus disturbed in mammalian HEK-293 cells.

The cationic C150–183 domain contains three repeated SPRRR motifs and specific phosphorylation of these serine residues (at positions S155, S162 and S170) could affect the RNA-binding to HBV-stC particles^21–23^. We exchanged the serine residues S155, S162 and S170 in the C150–183 domain with alanine residues (mimicking a non-phosphorylated state) or with glutamic acid and aspartic acid residues (mimicking a phosphorylated state) (Fig. 1f). This generated the pCI/stC_AAA_, pCI/stC_EEE_ and pCI/stC_DDD_ vectors, which were used to produce core particles in transiently transfected HEK-293 cells (Fig. 1f–h). Comparable with HBV-stC particles, HBV-stCEEE and HBV-stCDDD particles contained low amounts of cellular RNA (Fig. 1e,h). In contrast, purified HBV-stCAAA particles bound significant higher amounts of RNA than HBV-stC or mutant HBV-stCEEE and HBV-stCDDD particles (Fig. 1e,h; Supplementary Fig. S6). Silencing specific phosphorylation sites in the C150–183_AAA_ domain thus substantially enhanced the encapsidation of mammalian RNA into endogenously expressed particles *in vivo* in vector-transfected cells.

**Figure 2:**
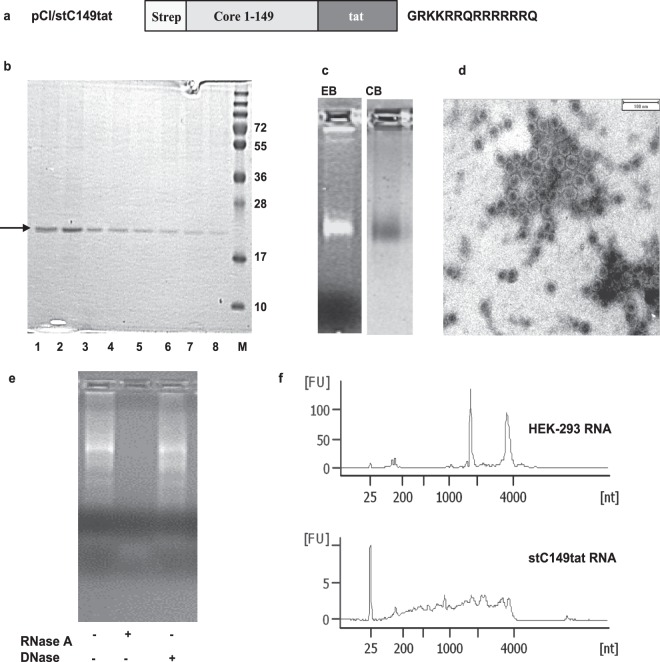
Expression and characterization of a mutant HBV-stC149tat antigen. (**a**) Schematic presentation of the HBV-stC149tat antigen. This antigen contained the HBV-stC149 sequence COOH-terminally fused with a 14-residue cationic HIV-tat_48–57_-like sequence. (**b–d**) 5 × 10^8^ HEK-293 cells were transiently transfected with the pCI/stC149tat vector. The HBV-stC149tat fusion protein was purified from cell lysates using StrepTactin sepharose-packed columns. (**b**) 10 µl of the elution fractions 1-8 were processed SDS-PAGE analysis followed by Coomassie Blue staining of the gel. Molecular weight marker (in kDa) is shown. (**c**) Fractions with the highest antigen content were pooled and processed for native agarose gel electrophoresis followed by ethidium bromide (EB) and subsequent Coomassie Blue (CB) staining of the gel. The original gel used to generate this cropped figure is shown in Supplementary Fig. S3a. Purified HBV-stC149tat antigen was further analyzed by electron microscopy (**d**). The indicated scale bar represents 100 nm. (**e**) Purified HBV-stC149tat particles were incubated with proteinase K and remained either untreated or treated with RNase A or DNase. Samples were subjected to native agarose gel electrophoresis followed by ethidium bromide (EB)-staining of the gel. (**f**) The length profile of HEK-293 RNA isolated from non-treated cells (upper panel) or particle-bound RNA (lower panel) was analyzed on a Bioanalyzer 2100.

To investigate whether the C150–183 sequence is crucial to bind mammalian RNA, we exchanged this domain with a short cationic HIV-tat_48–57_-like sequence (GRKKRRQRRRRRRQ; [www.uniprot.org/uniprot/P04610]) lacking any phosphorylation sites. This generated the pCI/stC149tat vector (Fig. 2a). The HBV-stC149tat antigen was isolated from transiently transfected HEK-293 cells with high purity, self-assembled into particles which captured high amounts of mammalian RNA (Fig. 2b–d; Supplementary Fig. S3a). This allowed us to analyze molecular and immune-stimulating features of the encapsidated RNA. Particle-bound nucleic acids were eliminated by RNase A but remained readily detectable after DNase I treatment (Fig. 2e), confirming the selective binding of RNA to HBV-stC149tat particles. Mammalian RNAs extracted from HBV-stC149tat particles varied in length from about 50 to 4000 nucleotides with no specific prevalence for small or large RNAs (Fig. 2f). The prominent ribosomal rRNA forms present in HEK-293 cells were not preferentially bound by HBV-stC149tat particles (Fig. 2f). This showed that HBV-stC149tat particles non-specifically captured mammalian RNAs via the cationic HIV-tat_48–57_-domain.

### Particle-bound mammalian RNA stimulates a Th1-biased core-specific humoral immune response by exogenous protein vaccination

**Figure 3:**
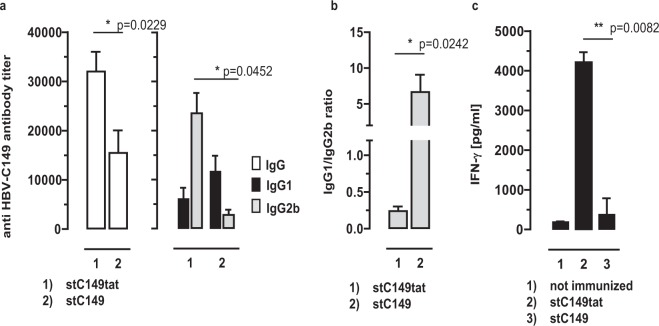
Induction of HBV core-specific antibodies in mice. (**a**) B6 mice were immunized with recombinant HEK-293-derived stC149tat or stC149 (n = 4/5). Three weeks post injection serum samples were obtained by tail bleeding and HBV core-specific IgG, IgG1 and IgG2b serum antibody titers were determined by end-point dilution ELISA using bacterial rHBV-C149 particles as detection antigen. Mean specific antibody titers in sera ±SD (**a**) and the calculated IgG1/IgG2a ratios ±SD (**b**) of a representative experiment (out of two performed experiments) are shown. The statistical significance of differences in IgG, IgG1 and IgG2b antibody titers between stC149tat- and stC149 immune B6 mice were determined by the unpaired Student’s t-test. (**c**) B6 mice were immunized with recombinant stC149tat or stC149 proteins. Ten days post immunization spleen cells were stimulated *ex vivo* for 2 days with the HBV-Core-specific I-A^b^-binding C128-140 peptide. The specific IFN-γ release into the cell culture supernatants was determined by ELISA. The statistical significance of differences in IFN-γ levels between stC149- and stC149tat-immune mice (groups 2 and 3) were determined by the unpaired Student’s t-test. (**a–c**) P values of <0.05 (*) and <0.01 (**) were considered statistically significant.

Bacterial RNA bound to recombinant HBV-C particles induced a core-specific, Th1-biased IgG2 antibody response in mice^16,17,19^. Similarly, a single injection of HBV-stC149tat particles (containing high amounts of mammalian RNA) preferentially induced core-specific Th1-biased IgG2b antibodies (IgG1/IgG2b ratios of ≤0.25) in C57Bl/6J (B6) mice (Fig. 3a,b). In contrast, RNA-free HBV-stC149 (Supplementary Fig. S4) preferentially induced IgG1 antibodies (IgG1/IgG2b ratio ≥5) (Fig. 3a,b). HBV-stC149tat antigen induced significant higher core-specific IgG antibodies than HBV-stC149 (Fig. 3a). The polarization of the core-specific immune responses was further confirmed by the inducible IFN-γ release of activated CD4^+^ T cells^16,17^. A prominent IFN-γ release was induced in T-cell preparations of HBV-stC149tat- but not HBV-stC149-immune or non-immunized mice upon *in vitro* stimulation with a well-defined I-A^b^-binding (C128–140; TPPAYRPPNAPIL) CD4^+^ T-cell epitope of HBV core (Fig. 3c), confirming that the antibody isotype profile is a reliable indicator for Th1/Th2*-*mediated immune responses in mice^24^.

**Figure 4:**
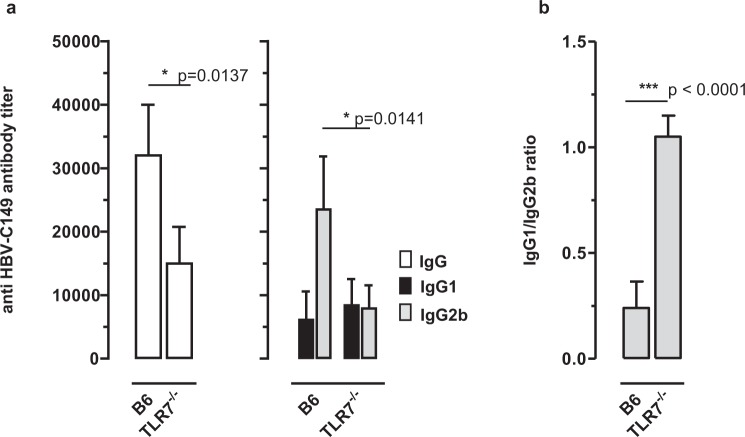
Induction of HBV core-specific antibodies in B6 and TLR7^−/−^ mice. B6 and TLR-7^−/−^ (n = 4/4) were immunized with recombinant HEK-293-derived stC149tat particles. Serum samples were obtained and analysed as described in the M&M section. Mean specific antibody titers in sera ±SD (**a**) and the calculated IgG1/IgG2a ratios ±SD (**b**) of a representative experiment (out of two performed experiments) are shown. The statistical significance of differences in IgG and IgG2b antibody titers between stC149tat immune B6 and TLR7^−/−^ mice was determined by the unpaired Student’s t-test. P values of <0.05 (*) and <0.001 (***) were considered statistically significant.

Bacterial RNA bound to recombinant HBV-C particles induced a Th1-biased IgG2 serum antibody response in TLR-7^+^ mice, but a decreased and balanced IgG1/IgG2 response in TLR-7^−/−^ mice^17^. Similarly, the IgG isotype profiles of the core-specific serum antibody responses induced by HBV-stC149tat particles differed in B6 and TLR-7^−/−^ mice (Fig. 4). A single injection of HBV-stC149tat particles induced higher core-specific IgG antibody titers in B6 mice than in TLR-7^−/−^ mice (Fig. 4a). HBV-stC149tat particles induced a balanced core-specific IgG1/IgG2b antibody response (IgG1/IgG2b ratios of 1 ± 0.15) in TLR-7^−/−^ mice (Fig. 4), but comparable Th1-biased antibody responses (IgG1/IgG2b ratios of ≤0.3) in TLR-3^−/−^ and B6 mice (Supplementary Fig. S7; Figs 3 and 4). In contrast, the HBV-stC149 vaccine induced core-specific Th2-biased IgG1 antibody responses (IgG1/IgG2b ratios of ≥5) in both, TLR-7^−/−^ and B6 mice (Supplementary Fig. S7; Fig. 3). Mammalian RNA captured by HBV-stC149tat particles thus stimulated a Th1-biased, TLR7-mediated humoral immunity by exogenous protein vaccination.

### Mammalian RNAs bound to non-particulate core antigens do not stimulate Th1-biased humoral immune responses by exogenous (protein) vaccination

**Figure 5:**
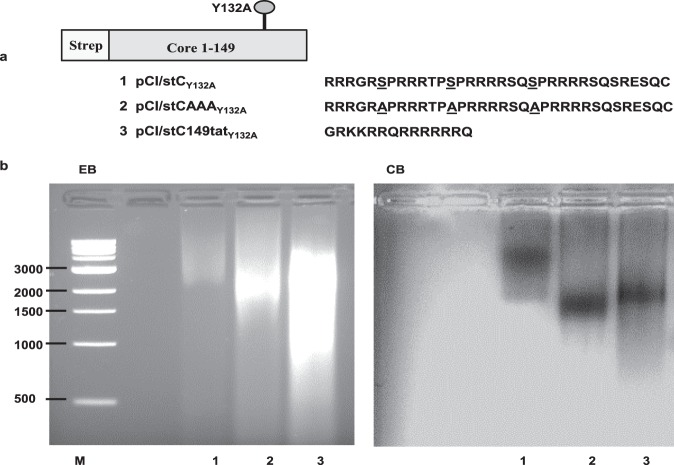
Expression and characterization of assembly deficient Core antigens. (**a**) Map of HBV-C antigens that contains a NH2-terminal strep tag sequence, an amino acid substitution from tyrosine to alanine at the position 132, and different COOH-terminal cationic domains: the wt stC_Y132A_, the stCAAA_Y132A_ (with additional substitutions of serine residues at positions S155, S162 and S170 to alanines) and stC149tat_Y132A_ (substitution of the wt C150-183 domain to a 14-residue HIV-tat like sequence). (**b**) Antigens were purified from cell lysates after transient transfection of 5 × 10^8^ HEK-293 cells with antigen encoding plasmids as described in the M&M. Identical amounts of recombinant antigens (2,5 µg; calculated for same amounts of monomers determined by SDS-PAGE) were processed for native agarose gel electrophoresis. 1 kb DNA ladder is shown. Agarose gel was stained with ethidium bromide (EB) followed by Coomassie Blue (CB) staining.

**Figure 6:**
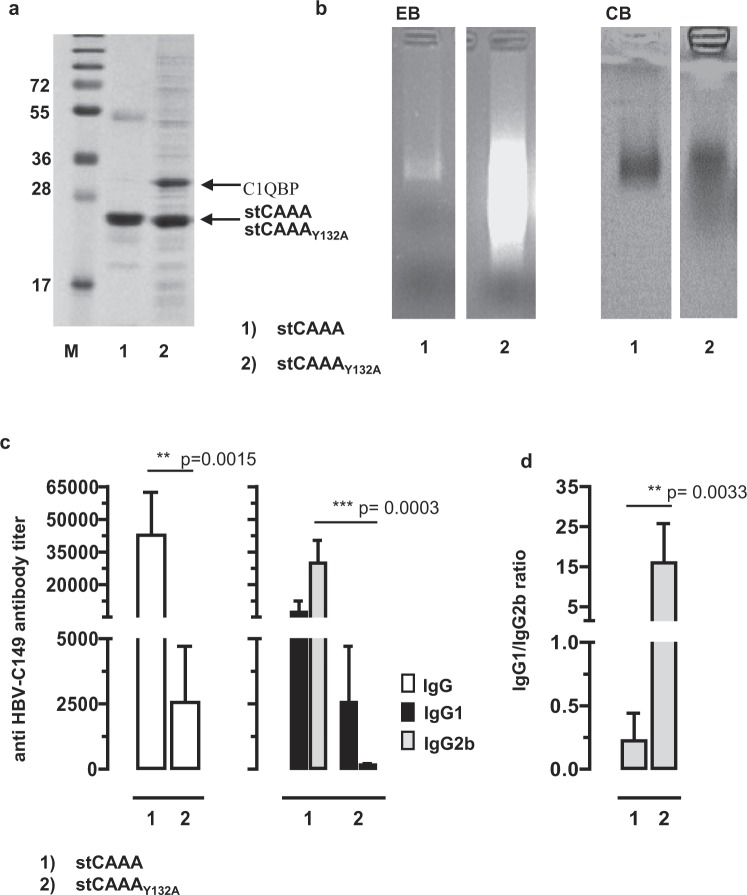
Expression and characterization of a mutant HBV-stCAAA and HBV-stCAAA_Y132A_ antigen. (**a**) Samples of purified stCAAA and stCAAA_Y132A_ antigens were processed for SDS-PAGE and subsequent Coomassie Blue staining of the gel. The positions of the respective Core antigens and the C1QBP co-precipitating with stCAAA_Y132A_ are indicated. The molecular weight marker in kDa is shown. (**b**) Same amounts of stC_AAA_ and the non-particulate stCAAA_Y132A_ (2 µg; calculated for same amounts of monomers determined by SDS-PAGE) were subjected to native agarose gels stained with ethidium bromide (EB) and subsequent with Coomassie Blue (CB). The original gel used to generate this cropped figure is shown in Supplementary Fig. S9. (**c**) B6 mice were immunized with recombinant HEK-293 derived stCAAA and stCAAA_Y132A_ antigens (n = 5/6). Serum samples were obtained 21 days post injection and analyzed for Core-specific IgG, IgG1 and IgG2b antibody titers by end-point dilution ELISA using bacterial rHBV-C149 as detection antigen. Mean specific antibody titers in sera ±SD of a representative experiment (out of two performed experiments) (**d**) and the calculated IgG1/IgG2a ratios ±SD are shown. (**c** and **d**) Statistically significant differences between the group 1 and group 2 were determined using the unpaired student’s t-test. P values of <0.01 (**) and p < 0.001 (***) were considered statistically significant.

We next asked if encapsidation of mammalian RNA into particles is a prerequisite to elicit Th1-biased antibody responses by exogenous protein vaccination and if freely exposed cationic domains in assembly-deficient core antigens have an impact on the net RNA binding of mutant core antigens. Therefore, we constructed assembly-deficient HBV core variants encoding different cationic domains (C150–183, C150–183_AAA_ or HIV-tat_48–57_) by exchanging the thyrosine (Y) at position 132 with an alanine (A) residue^25^. This generated the pCI/stC_Y132A_, pCI/stCAAA_Y132A_ and pCI/stC149-tat_Y132A_ vectors, respectively (Fig. 5a). All antigens were efficiently expressed in transiently transfected HEK-293 cells and we could not detect particulate structures in any of these preparations by electron microscopy (data not shown). Non-particulate antigens showed a clear increase in the RNA binding between stC_Y132A_ and stCAAA_Y132A_ and between stCAAA_Y132A_ and stC149-tat_Y132A_ (Fig. 5b). This confirmed above findings that prevention of specific phosphorylation in cationic C150–183 domain and, in particular, the non-phosphorylatable cationic tat domain enhanced binding and encapsidation of cellular RNAs into particles (Figs 1 and 2). However, non-particulate antigens were associated with cellular proteins that co-purified in our expression system (Fig. 6a; Supplementary Fig. S8). In particular, the HBV-stC149tat_Y132A_ antigen with the highest RNA binding capacity (Fig. 5b) stable bound high amounts of cellular proteins (Supplementary Fig. S8).

To directly compare the RNA-binding and immunogenicity of non-particulate and particulate antigens, we used HBV-stCAAA_Y132A_ and HBV-stCAAA antigens showing intermediate RNA-binding (Figs 1 and 5). The non-particulate HBV-stCAAA_Y132A_ bound 5–10 fold higher amounts of mammalian RNA than HBV-stCAAA particles (calculated for the same amount of monomers), accompanied with an enhanced binding of cellular proteins (Fig. 6a,b; Supplementary Fig. S9). This suggested that the co-purified cellular proteins bind to cellular RNAs captured by the mutant cationic domain in the non-particulate HBV-stCAAA_Y132A_ antigen. MALDI-TOF-MS analyses (commercially performed by Toplab Corp., Planegg, Germany) identified the most prominent protein co-precipitating with HBV-stCAAA_Y132A_ as the 32 kDa mature form (residues 74–282) of the complement component 1 Q subcomponent-binding protein (C1QBP) also called gC1qR or HABP1 (Fig. 6a; Supplementary Fig. S10b)^26^. C1QBP binds to the cationic C150–183 domain of a monomeric HBV-P22 precore protein but not to particulate HBV-C protein^27^. RNase A treatment of HBV-stCAAA_Y132A_ preparations quantitatively destroyed the bound RNA and removed most of the co-precipitated cellular proteins (Supplementary Fig. S10). The binding of the C1QBP protein to mutant HBV-stCAAA_Y132A_ was resistant to RNase A treatment (Supplementary Fig. S10b), indicative for a protein-protein interaction.

To evaluate the antigenicity of particulate and non-particulate RNA-binding antigens, we vaccinated mice with the same amounts of recombinant HBV-stCAAA particles or HBV-stCAAA_Y132A_ antigen (calculated for the same amount of monomers). HBV-stCAAA particles induced significant higher core-specific IgG antibodies than non-particulate HBV-stCAAA_Y132A_ antigen (Fig. 6c). Comparable to HBV-stC149-tat particles, HBV-stCAAA particles induced a core-specific Th1-biased antibody response in B6 mice (Figs 3 and 6c,d), demonstrating that the vaccine-induced Th1-immunity was independent from the cationic motif and therefore primarily depends on the particle-bound RNA. In contrast, the non-particulate HBV-stCAAA_Y132A_ antigen, while binding higher amounts of RNA than HBV-stCAAA particles, induced an almost exclusive Th2-biased IgG1-specific antibody response (IgG1/IgG2b ratios of ≥15; Fig. 6c,d). Encapsidation of cellular RNA into particles thus facilitated induction of a Th1-biased humoral immunity in mice by exogenous protein immunization.

### Murine RNAs captured by endogenously expressed particles or non-particulate core antigens stimulate a Th1-biased humoral immunity by DNA vaccination

**Figure 7:**
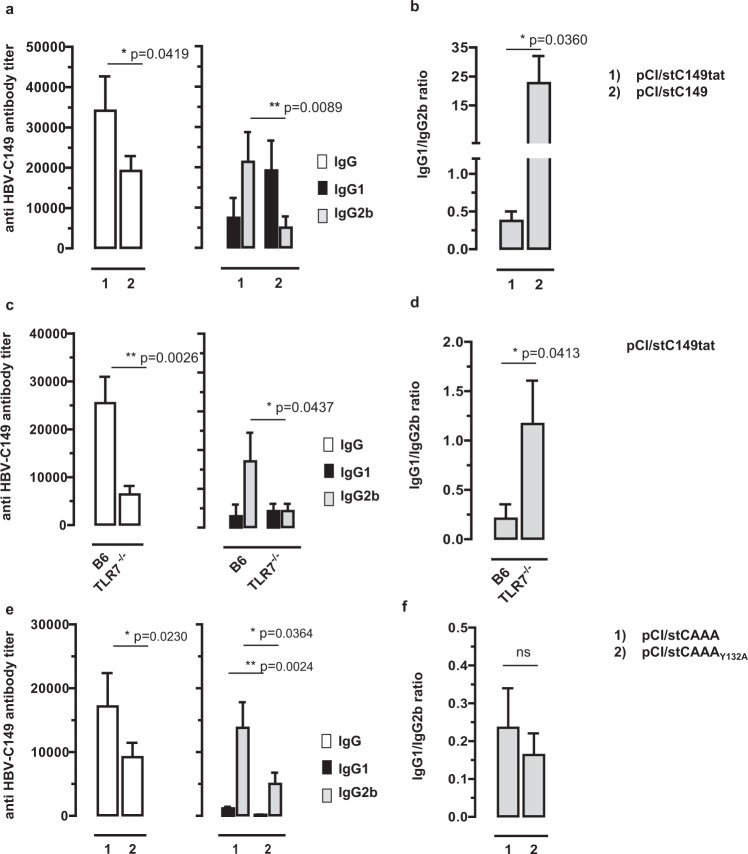
Characterization of antibody responses induced by DNA vaccines expressing particulate and non-particulate core antigens. (**a**–**f**) Mice were immunized intradermally with 2 μg particle-coated plasmids with the gene gun (see Supplemental protocols). At d21 mice were boosted with the same vectors. The specific serum Ab responses and isotype profiles (IgG, IgG1, IgG2a) were determined 12 days post boost immunization by end-point dilution ELISA using bacterial rHBV-C149 particles as detection antigen and IgG1/IgG2a ratios were calculated. (**a**,**b**) B6 mice (n = 3/4) were immunized with pCI/stC149tat or pCI/stC149 vectors. (**c**,**d**) B6 and TLR7^−/−^ mice (n = 3/5) were immunized with pCI/stC149tat. (**e,f**) B6 mice (n = 5/5) were immunized with pCI/stCAAA or pCI/stCAAA_Y132A_ plasmid DNA. Mean specific antibody titers in sera (**a**,**c**,**e**) and the calculated IgG1/IgG2a ratios ±SD (**b,d,f**) of representative experiments (out of two experiments performed) are shown. Where indicated, the statistical significance of differences in IgG, IgG1 and IgG2b antibody titers was determined by the unpaired Student**’**s t-test. P values of < 0.05 (*) and < 0.005 (**) were considered statistically significant.

Endogenously expressed HBV-C, but not HBV-C149 and HBV-E induced a Th1-biased humoral immunity in H-2^d^ BALB/c mice vaccinated with low amounts (1–2 µg) of vector DNA with the gene gun^16^. Similarly, the pCI/stC149tat and pCI/stC149 vectors induced Th1- (IgG1/IgG2b ratios of <0,4) and Th2-biased core-specific antibody responses (IgG1/IgG2b ratios of >10) in H-2^b^ B6 mice, respectively and the total core-specific IgG response was significantly enhanced in pCI/stC149tat-immune B6 mice (Fig. 7a,b). Furthermore, the pCI/stC149tat vector induced significant higher levels of core-specific IgG antibodies in B6 mice as compared to TLR-7^−/−^ mice (Fig. 7c). Most interestingly, the pCI/stC149tat vector induced a balanced core-specific IgG1/IgG2b antibody profile (IgG1/IgG2b ratios of 1 ± 0.4) in TLR-7^−/−^ mice (Fig. 7c,d). This showed that murine RNA directly captured in host APCs by endogenously expressed HBV-stC149tat particles operates as a TLR-7 ligand and stimulates a Th1-biased humoral immunity.

Furthermore, pCI/stCAAA and pCI/stCAAA_Y132A_ vectors, expressing RNA-binding particulate and non-particulate antigens, primed Th1-biased core-specific antibody responses (IgG1/IgG2b ratios of ≤0.35) in B6 mice (Fig. 7e,f). The pCI/stCAAA_Y132A_ vector expressed 2-3 fold higher steady state levels of core antigen than the pCI/stCAAA vector in transiently transfected cells (Supplementary Fig. S11), but endogenously expressed stCAAA particles induced higher core-specific IgG antibody titers than the non-particulate stCAAA_Y132A_ antigen (Fig. 7e). However, and in contrast to protein vaccines, the pCI/stCAAA_Y132A_ vector expressing the non-particulate stCAAA_Y132A_ antigen induced a Th1-biased core-specific antibody response (IgG1/IgG2b ratios of ≤0.25) (Figs 6c,d and 7e,f). Thus, the non-particulate stCAAA_Y132A_ antigen induced a Th1-biased core-specific antibody response by endogenous (DNA) but not exogenous (protein) vaccines.

## Discussion

Chimeric HBV core particles displaying foreign epitopes or antigenic domains on their surface emerged as a platform technology for the induction of humoral immune responses by recombinant protein-based vaccines^28^. We here show novel features of HBV core antigen for its usage in DNA-based vaccines: First, different cationic domains located COOH-terminally to the same C1–149 antigen backbone non-specifically bound different amounts of mammalian RNAs in transiently transfected cells, and RNA-binding to both, particulate and non-particulate antigens was significantly enhanced by silencing and/or omitting specific phosphorylation sites in these domains. Second, mammalian RNAs encapsidated in HBV core particles, but not bound to non-particulate core antigens stimulated TLR-7-mediated, Th1-biased core-specific humoral immune responses by exogenous protein immunization. Third, endogenously expressed HBV core particles as well as non-particulate core antigens capture murine RNAs in *in vivo* transfected APCs that stimulate Th1-biased core-specific humoral immune responses by DNA immunization. We could show that induction of Th1-biased humoral immune responses  by endogenously expressed RNA-binding particles critically depends on TLR-7 signaling. Mammalian RNAs targeted by an endogenously expressed vaccine antigen thus function as an endogenous adjuvant^11–13^ in the host, which facilitates priming of Th1-biased immune responses by DNA-based immunization.

The 183-residue HBV-C contains two well-defined domains: The NH2-terminal 149 aa comprise an assembly domain sufficient for particle formation and a COOH-terminal cationic domain (aa 150–183) that mediates binding of HBV-C to nucleic acids, in particular pregenomic RNA (pgRNA) in HBV-infected cells. Specific binding of viral pgRNA to HBV-C in an infected cell requires concerted interactions between the cationic HBV-C domain, the viral polymerase and a 5′ ε stem-loop in the pgRNA^29–31^. However, the mechanism(s) preventing the non-specific binding of cellular RNAs to the cationic C150–183 domain of HBV-C in HBV-infected cells are largely unknown. Non-specific binding of RNA may depend only on interactions between the positively charged, arginine-rich C150–183 domain and negatively charged RNA molecules. Synthetic HBV-C derived peptides C150–157 (RRRDRGRS) or C164–179 (SPRRRRSQSPRRRRSQ) tightly bound nucleic acids *in vitro*, indicating that the guanidinium head groups on the arginine-residues interact with the negatively charged phosphate backbone of RNA^32,33^. We here showed that identical HBV-stC particles bind low amounts of mammalian RNA, but high amounts of bacterial RNA (Fig. 1e; Supplementary Fig. S3). Non-specific incorporation of mammalian RNA into particles, but also binding of RNA to non-particulate core antigens in transfected cells was significantly enhanced by preventing the specific phosphorylation of the cationic domain, either by exchanging the entire cationic C150–183 domain with a cationic HIV-tat_48-57_-like domain (HBV-stC149tat) or by exchanging the serine residues at positions S155, S162 and S170 with alanine residues (HBV-stC_AAA_). It has been shown that the phosphorylation state of the cationic C150-183 domain, and in particular of the serine residues S155, S162 and S170 (or S157, S164 and S172 in the genotype A), is crucial for the specific encapsidation of viral pgRNA into HBV-C particles, reverse transcription and intracellular trafficking of capsids^21,23,34–36^. When these serines were exchanged with alanine residues viral pgRNA was not encapsidated into HBV-C particles^21,37^. This suggested that both, non-specific binding of cellular RNAs and specific binding of pgRNA to endogenously expressed HBV core antigens *in vivo* is regulated by the phosphorylation state of the C150–183 domain.

‘Exogenous’ bacterial^17^ and mammalian RNA bound to isolated HBV core particles functioned as TLR-7 ligand and stimulated Th1-biased antibody responses in mice. Usually, TLRs are not directly stimulated by microbial or mammalian nucleic acids, because their ligand-binding domains face into the lumen of the endo/lysosomal compartment^5^. Therefore, TLR-stimulating nucleic acids must be translocated from the cytosol and/or from the outside of a cell into the endo/lysosomal compartment. Disruption of core/RNA particles prevented the Th1-inducing adjuvant effect of bacterial RNA^16^. Similarly, a non-particulate HBV-stCAAA_Y132A_ antigen, while binding significant higher amounts of mammalian RNA than its particulate HBV-stCAAA form elicited a Th2-biased humoral immunity by protein vaccination. This indicated that encapsidation of heterologous RNA into HBV-C particles is crucial to protect the immune-stimulating RNA from rapid degradation after injection into mice and/or to deliver it into the TLR-7 expressing endo/lysosomal compartment of APCs. Similarly, antigen-encoding RNA condensed with a cationic protamine peptide stimulate TLR-7-dependent, Th1-biased adaptive immune responses by RNA-based vaccines^38,39^. Several virus-specific RNA motifs and polyuridylic (polyU) sequences that engage the TLR-7 receptor have been identified^40^. HBV-stC149-tat particles contained mammalian RNAs that varied in length from about 50 to 4000 nucleotides, but it is unknown whether a specific RNA species and/or specific motifs within these RNA molecules engage the TLR-7. It has been shown that B-cells specifically take up recombinant HBV-C particles^17^. Specific protein spikes on the surface of HBV-C particles could cross-link membrane-bound immunoglobulin on B-cells^17^. RNA-bound HBV-Core particles could activate B-cells by combined B-cell antigen receptor/TLR-7 engagement^7,41^. This could further explain the exceptional potent immunogenicity of exogenous RNA-bound HBV-C particles to trigger Th1-biased humoral immune responses. Non-particulate core antigens present with different cationic domains bound significant higher amounts of mammalian RNA, but also cellular proteins than their particulate forms. This suggested that freely exposed cationic domains in non-particulate antigens efficiently bound cellular RNA. However, we cannot exclude (and prevent) that the bulk of cellular RNA as well as cellular proteins stable bind to these non-particulate antigens during cell extraction. Thus, these ‘dirty’ non-particulate antigens are not attractive for their usage as recombinant vaccines.

Endogenously expressed intracellular or secreted antigens, e.g., HBV-surface, HBV-C149 and HBV-E that do not bind cellular RNA induced a Th1-biased humoral immunity in mice when vaccinated with high amounts (50–100 µg) of naked vector DNA, but a Th2-biased humoral immunity when vaccinated with low amounts (1–2 µg) of plasmid DNA by intradermal delivery with the gene gun^16,42,43^. This confirmed that high amounts of plasmid DNA itself, for example via binding to endogenous DNA sensor(s) that activate the TBK1-STING pathway^5^ induce Th1-stimulating innate signals in mice^42^. The only exception we have observed up to now was the priming of specific Th1-biased responses by gene gun vaccination of DNA expressing RNA-capturing core antigens (Fig. 7)^16^. Most interestingly, particle-bound RNA stimulated a Th1-biased immunity by both, protein and DNA vaccination, whereas RNA bound to non-particulate antigens almost exclusively induced Th2-biased immune responses by exogenous (protein) antigens and Th1-biased immune responses by endogenous (DNA) antigens. Furthermore, the total core-specific IgG antibody responses were significantly lower in mice vaccinated with RNA-bound non-particulate antigens either as protein or DNA vaccine. This indicated that core-specific antibodies directed against an immunodominant c/e1 epitope presented on the tip of protein spikes on the surface of HBV-C particles (i.e., the major immunodominant region between C78–82; MIR)^44^, were efficiently induced by particulate but not non-particulate antigens^16^. Neither HBV-Core particles nor non-particulate antigens used in this study contain transmembrane domains or enter the secretory pathway [www.uniprot.org/uniprot/P03146] and therefore are intracellular antigens. It is unclear how DNA vaccines prime antibody responses to intracellular antigens as B-cells require exogenous antigen for stimulation. Therefore, at least a small amount of antigen must be released from *in vivo* transfected antigen-expressing cells, for example, induced by cell death mechanisms^3^. However, the priming of Th1-biased antibody responses to RNA-bound non-particulate core antigens by DNA but not protein vaccines suggested that different mechanisms, local milieus and/or cell types are involved in antigen/RNA uptake by B-cells.

In this study, we focused on the induction of humoral immune responses by endogenously expressed core variants differing in particle formation and RNA binding. However, endogenously expressed RNA-capturing HBV-C particles also efficiently induced CD8^+^ T-cell responses and controlled HBV clearance in murine infection model^45–47^. Similarly, we could induce HBV-C but not HBV-surface- specific effector CD8^+^ T-cell responses in 1.4HBV-S^mut^ tg mice that harbor a replicating HBV genome in hepatocytes by DNA-based vaccination^48–50^. HBV-C (K^b^/C93)-specific CD8^+^ T-cells accumulated in the liver of pCI/C-immune 1.4HBV-S^mut^ tg mice and suppressed (at least transiently) HBV DNA replication^49,50^. 1.4HBV-S^mut^ tg mice express endogenous HBV-C and circulating HBV-E antigens in the liver^48^. Antiviral HBV-C-specific CD8^+^ T-cells are therefore induced under stringent conditions (i.e., operating against the tolerogenic milieu of an antigen expressing liver and the tolerogenic features of the circulating HBV-E in the blood)^51^ by DNA-based immunization.

In summary, targeting an endogenous RNA adjuvant activity in vaccine recipients by antigens expressing well-defined cationic domains may help to design new generations of DNA vaccines that efficiently prime Th1-biased antibody and T-cell responses against chronic virus infections or tumours^52,53^.

## Materials and Methods

### Mice

C57BL/6J (B6) (Janvier, France), TLR-3^−/−^ (Jackson, no. 009675), TLR-7^−/−^ (Jackson, no. 008380) were bred and kept under standard pathogen-free conditions in the animal colony of Ulm University. All mouse immunization studies were carried out in strict accordance with the recommendations in the Guide for the Care and Use of Laboratory Animals of the German Federal Animal Protection Law. The protocols were approved by the Committee on the Ethics of Animal Experiments of the University of Ulm (Tierforschungszentrum Ulm, Oberberghof) and the Regierungspräsidium Tübingen (Permit Number 992 and 1231 to RS). All immunizations were performed under short time Isofluran anesthesia and all efforts were made to minimize suffering.

### Expression of HBV-stC particles in HEK-293 cells

For large scale production of HBV core antigens, we transfected 5 × 10^8^ (80–100 dishes) HEK-293 cells with the indicated plasmids using the calcium phosphate method. HEK-293 cells were used because they can be transfected with high efficacy (≥90%) using the calcium phosphate method and express high levels of vector-encoded antigens (data not shown). 48 hours after transfection, cells were lysed with pH 8.0 lysis buffer [150 mM NaCl, 0.5% NP40 and 100 mM Tris-hydrochloride supplemented Protease Inhibitor Cocktail Tablets (cat. no. 11836145001, Roche Applied Science, Penzberg, Germany)]. Notably, different cell lysis procedures (e.g., CHAPS-detergent containing buffers or detergent-free homogenization)^20^ can be used in this step that all preserved the particle/RNA complexes. Extracts were cleared by centrifugation and purified using the Strep-tag purification system. Briefly, cell extracts were passed over StrepTactin sepharose (cat. no. 2-1201-025; IBA, Göttingen, Germany) packed polypropylene columns (cat. no. 29922; Pierce, Rockford, USA), purified with five column volumes of wash buffer [150 mM NaCl, 1 mM EDTA and 100 mM Tris–hydrochloride (pH 8.0)] and eluted in eight 500 μl fractions in elution buffer [30 mM NaCl, 0.2 mM EDTA and 20 mM Tris–hydrochloride (pH 8.0) supplemented with 2.5 mM desthiobiotin (cat. no. 2-1000-002; IBA, Göttingen, Germany)]. Where indicated, samples were concentrated in a Speed Vac Concentrator to a volume of 100 μl. Protein samples were analysed on 12.5% SDS-PAGE gels in a Tris-Glycine buffered system and stained with Coomassie blue or on standard 1.2% agarose gels stained with ethidium bromide followed by Coomassie Blue staining of the gels.

### Characterization of particle-bound RNA

RNA was extracted from purified particles using RNAmagic reagent (cat. no.: 56-1000-100; Bio-Budget Technologie, Krefeld, Germany) according to the manufacturer’s instructions followed by a further clean-up step with a RNeasy mini kit (cat. no. 74104; Qiagen, Hilden, Germany). RNA samples were directly used for Bioanalyzer (2100 Expert; Agilent Technologies, Inc. Waldbronn, Germany) measurements with the RNA 6000 Nano Kit (cat. no. 5067-1511, Agilent Technologies, Waldbronn, Germany).

### Immunization of mice

Mice were immunized intramuscularly (*tibialis anterior* muscles) with 100 μg plasmid DNA or subcutaneously with indicated amounts of recombinant HBV core antigens in Alhydrogel, kindly provided by Dr. Erik Lindblad (Brenntag Biosector, Frederiksund, Denmark). Gene gun immunization was performed as described in Supplementary Protocols.

### Determination of serum antibody titers

Serum samples were obtained by tail bleeding. Murine IgG, IgG1 and IgG2b serum antibody titers were determined by end-point dilution ELISA using bacterial HBV-C149 or -C particles as detection antigens as described previously^16^. Briefly, microELISA plates (Nunc-Maxisorp, Wiesbaden, Germany) were coated with 150 ng rAgs/well in 50 μl 0.1 M sodium carbonate buffer (pH 9.5) at 4 °C. Serial dilutions of the sera in loading buffer (PBS supplemented with 3% BSA) were added to the Ag-coated wells. Serum Abs were incubated for 2 h at 37 °C followed by fife washes with PBS supplemented with 0.05% Tween 20. Bound serum Abs were detected using HRP-conjugated anti-mouse IgG Abs (catalog no. 554002; BD PharMingen, Hamburg, Germany), IgG1 (catalog no. 559626; BD PharMingen, Hamburg, Germany) and IgG 2b (catalog no. 610-4342; Rockland; Limerick, PA USA) at a dilution of 1/2000 followed by incubation with *o*-phenylenediamine × 2 HCl in substrate buffer [50 mM Na_2_HPO_4_, 25 mM citric acid (pH 5.0)]. The reaction was stopped by 1 M H_2_SO_4_ and the extinction was determined at 492 nm. End-point titers were defined as the highest serum dilution that resulted in an absorbance value three times greater than that of negative control sera (derived from non-immunized mice).

### Statistics

Data were analyzed using PRISM software (GraphPad, San Diego, CA). The statistical significance of differences in the mean specific antibody titers in sera ±SD and the calculated IgG1:IgG2a ratios ± SD of different groups were determined by the unpaired student’s t-test. P values < 0.05 (*) were considered statistically significant (** significant at p < 0.01, *** significant at p < 0.001).

## Electronic supplementary material

Supplementary information

## Data Availability

The data sets generated in this study are available from the corresponding author upon reasonable request.
