# Supplementary material for: Cationic domains in particle-forming and assembly-deficient HBV core antigens capture mammalian RNA that stimulates Th1-biased antibody responses by DNA vaccination
Source: Sci Rep. 2018 Oct 2;8:14660. doi: 10.1038/s41598-018-32971-5 (PMC6168482; doi:10.1038/s41598-018-32971-5)
Supplement: Supplementary file 1 — Supplementary information [file 41598_2018_32971_MOESM1_ESM.pdf]

Supplementary Figures &
Supplementary Protocols

Cationic domains in particle-forming and assembly-deficient HBV core antigens capture mammalian RNA that stimulates Th1-biased antibody responses by DNA vaccination

Jana Krieger[1], Katja Stifter[1], Petra Riedl[1] and Reinhold Schirmbeck[1*]

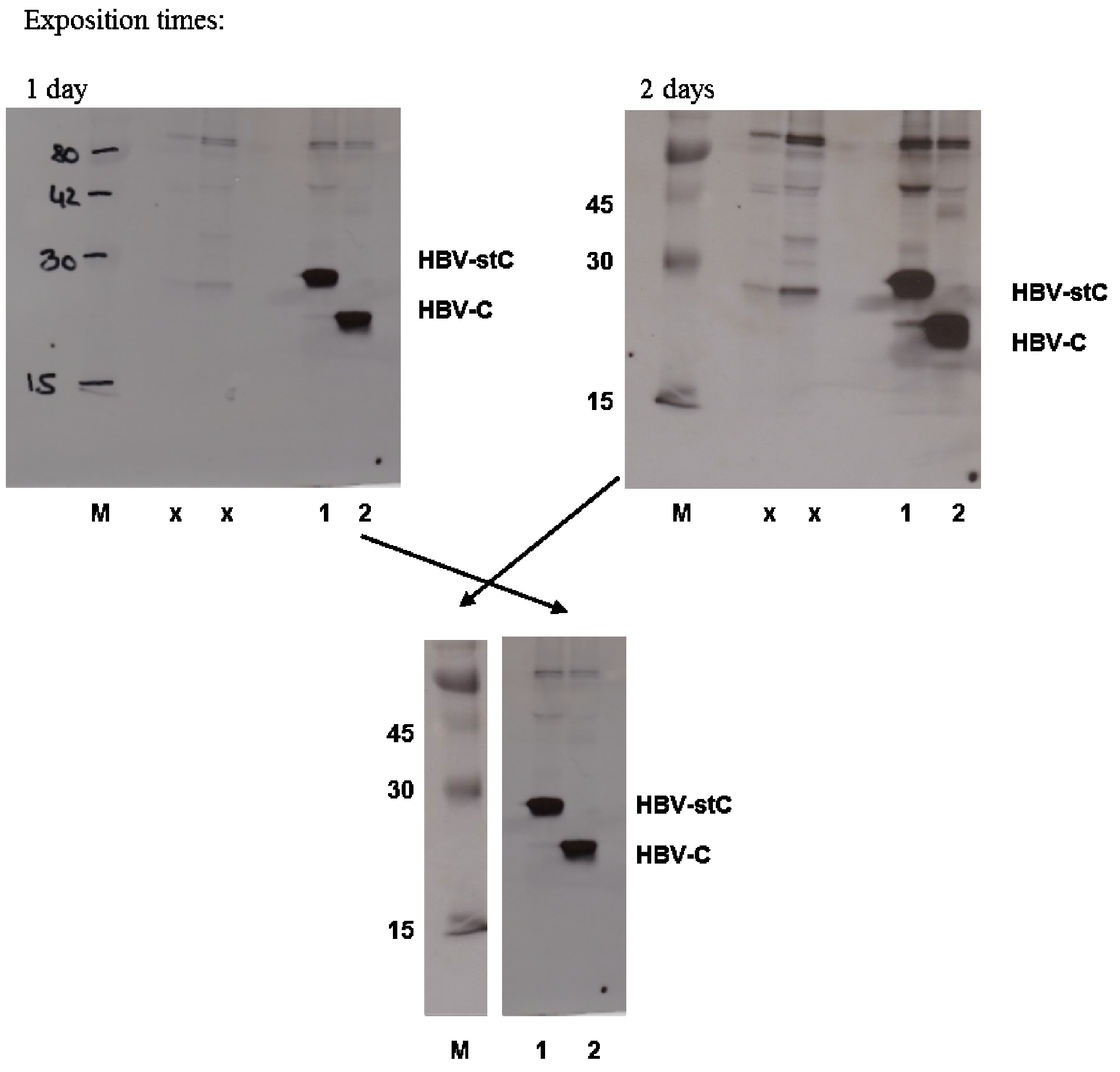

Supplementary Fig. S1

Generation of cropped Figure 1b. (a) HEK-293 cells were transiently transfected with pCI/stC (lanes 1) or pCI/C (lanes 2) and labelled with ^{35}S-methionine/cysteine. Cell lysates were immunoprecipitated with a polyclonal rabbit anti HBV-C serum and processed for SDS-PAGE and fluorography of the gels. Due to weak intensity of the marker bands after d1 of exposition of the gel, the marker lane was taken from a longer exposed (d2) fluorography. x: samples not relevant for this manuscript. The positions of HBV-stC and HBV-C are indicated.

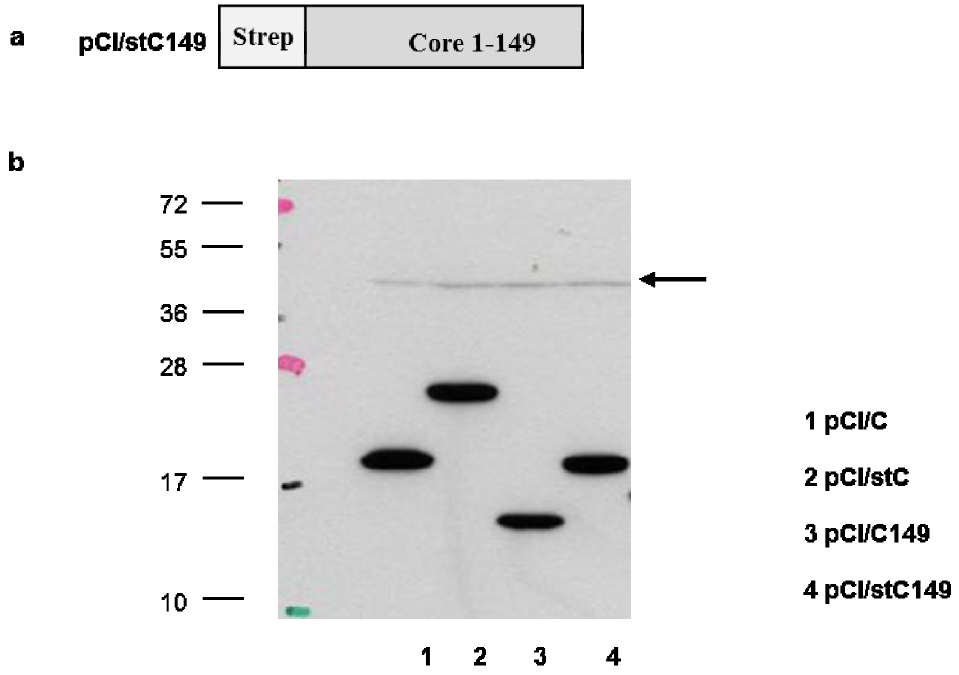

Supplementary Fig. S2

Western blot analysis of HBV core antigens. (a) Schematic presentation of the truncated stC149 lacking the cationic C150-183 domain. **(b)** HEK-293 cells were transfected with the indicated vectors (lane 1¸ pCI/C; lane 2, pCI/stC; lane 3, pCI/C149; lane 4 pCI/stC149), lysed and directly processed for SDS-PAGE analysis followed by Westen Blotting using a polyclonal rabbit anti Core serum and anti-actin antibody. The beta-actin band is marked by an arrow. Molecular weight marker (in kDa) is given.

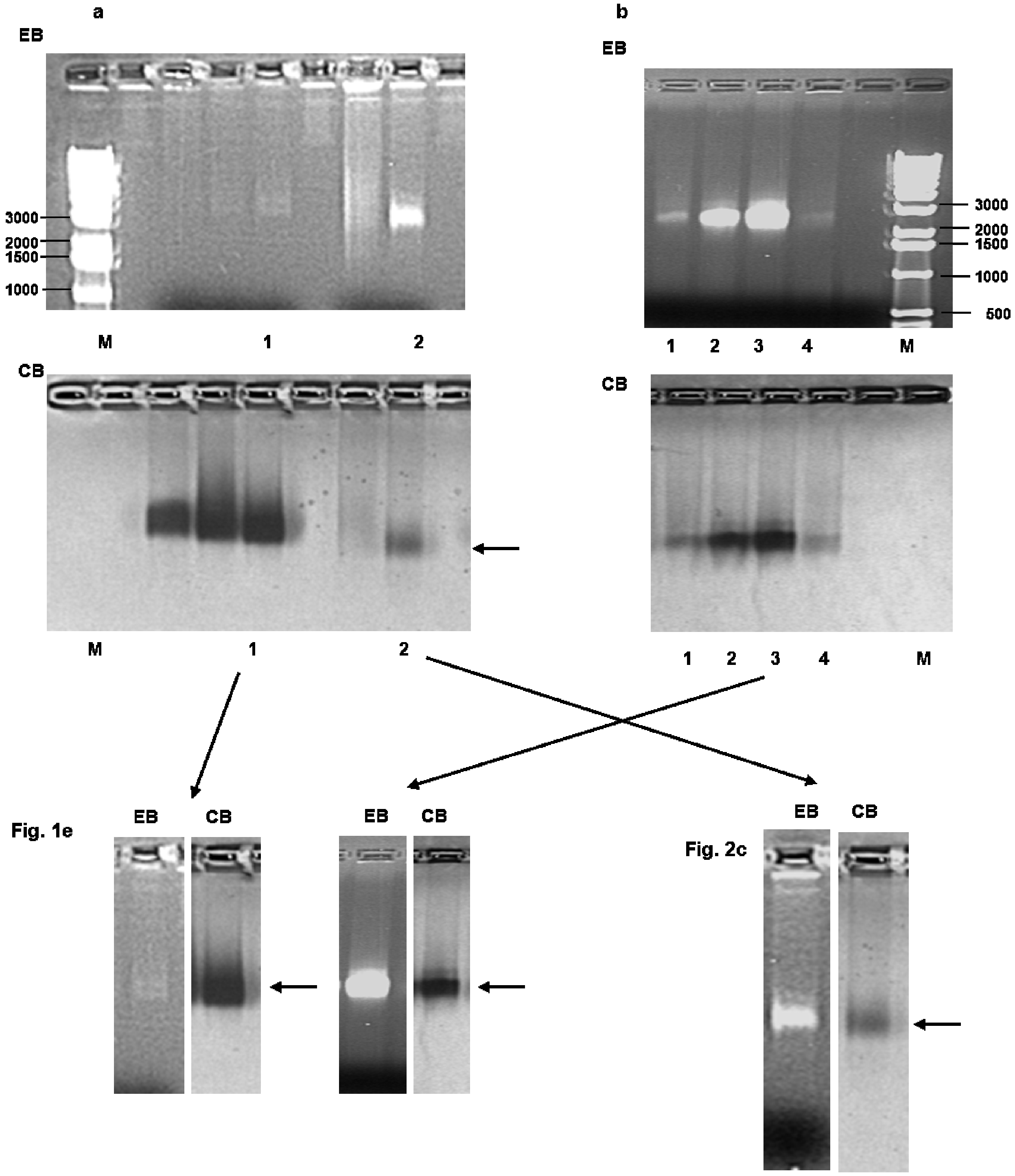

Supplementary Fig. S3

Generation of cropped Figures 1e and 2c. Samples of **(a)** purified HBV-stC (lanes 1) and HBV-stC149tat antigens (lanes 2) expressed in HEK-293 cells and **(b)** HBV-stC antigen (lanes 1-4) expressed in bacteria were run on individual agarose gels, followed by ethidium bromide (EB) and subsequent Coomassie Blue (CB) staining of the gels. Arrows indicate the origin of HBV-stC expressed in HEK-293 cells (**a;** lanes 1) and bacteria (**b;** lanes 3) shown in Figure 1e (lower panel), and the HBV-stC149tat expressed in HEK-293 cells (**a**; lanes 2) shown in Figure 2c (lower panel).

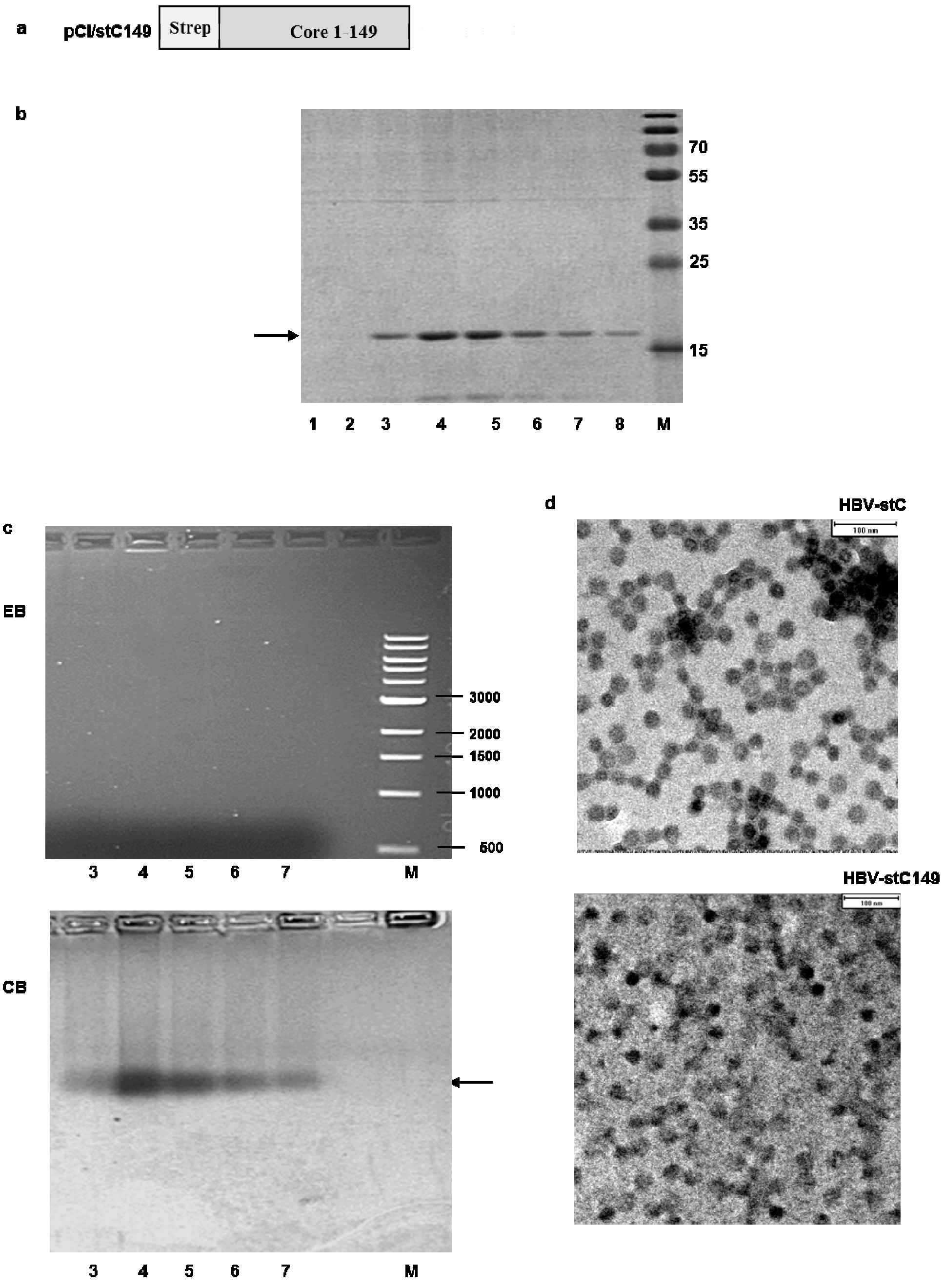

Supplementary Fig. S4

Expression and characterization of a truncated HBV-stC149 antigen. (a) Schematic presentation of the truncated stC149 lacking the C terminal domain. **(b)** 5 x 10^8 HEK-293 cells were transiently transfected with the pCI/stC149 vector. The HBV-stC149 fusion protein was purified from cell lysates using StrepTactin sepharose-packed columns and eluted in 8x 500 µl fractions. 10 µl samples were processed for SDS-PAGE analysis followed by Coomassie Blue staining of the gel. **(c)** Fractions 3 to 7 were concentrated and processed for agarose gel electrophoresis followed by ethidium bromide (EB) and Coomassie Blue (CB) staining of the gels. **(d)** Electron microscopy images of purified HBV-stC and HBV-stC149 antigens. The indicated scale bar represents 100nm.

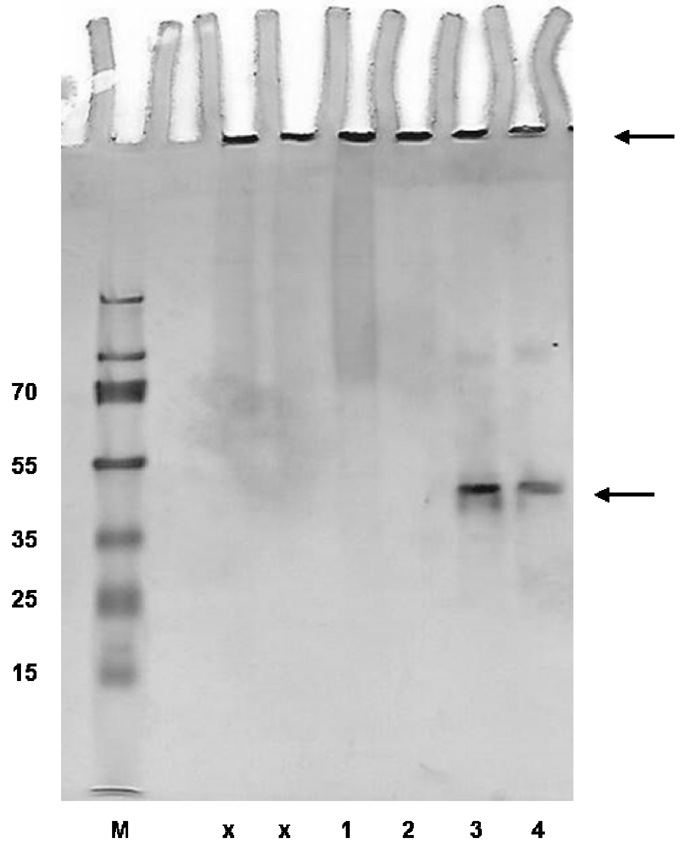

Supplementary Fig. S5

Determination of particle formation in HBV-stC and HBV-stC149 preparations. Purified HBV-stC (lanes 1 and 2) and HBV-stC149 (lanes 3 and 4) preparations were run on SDS-PAGE gels under non-reducing conditions (without mercaptoethanol) followed by Coomassie blue staining of the gel. The positions of the particulate antigens (not migrating into the gel) and a proportion of non-particulate HBV-stC149 antigen are indicated by arrows. The molecular marker (in kDa) is shown. x: samples not relevant for this manuscript

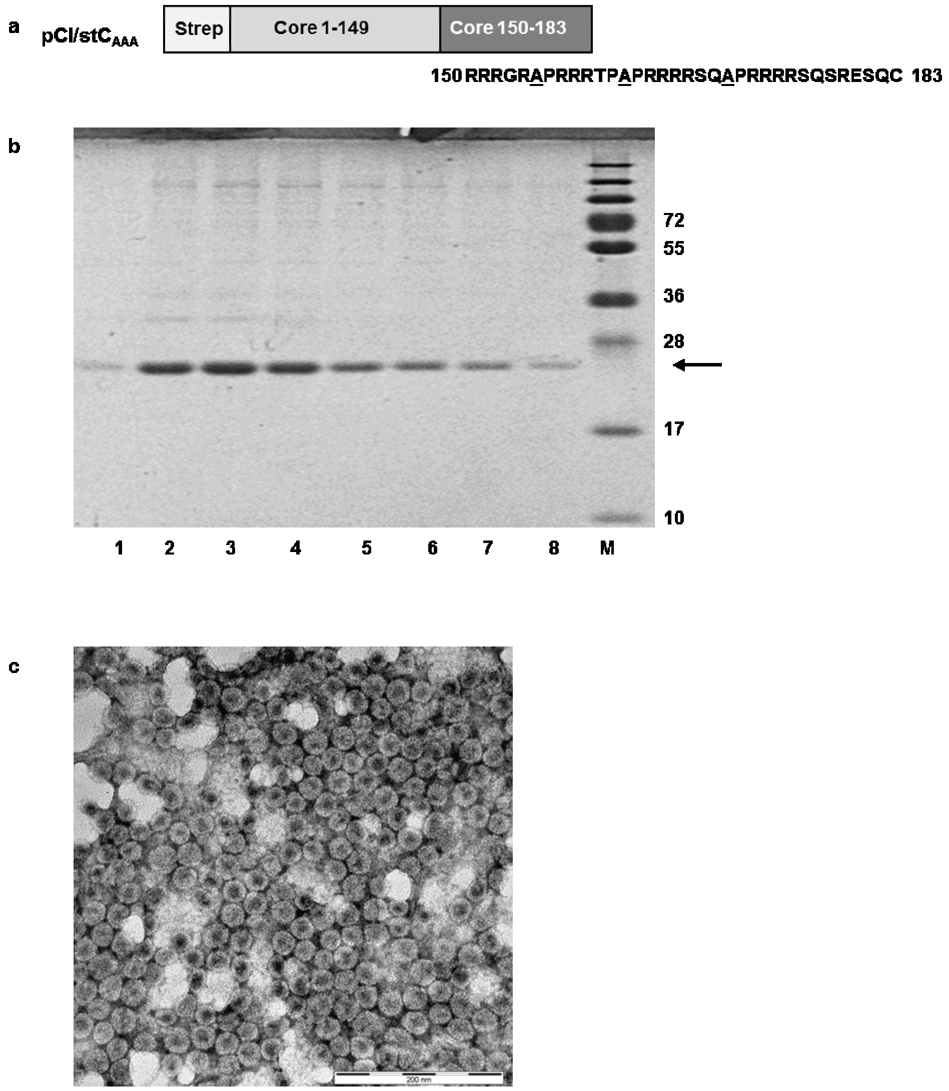

Supplementary Fig. S6

Expression and characterization of a mutant HBV-stC$_{AAA}$ antigen. (**a**) Schematic presentation of the mutant HBV-stC$_{AAA}$. The substitution of serine residues at positions S155, S162 and S170 to alanine are indicated. **(b)** HEK-293 cells were transiently transfected with the pCI/stC$_{AAA}$ vector. The antigen was purified from cell lysates using StrepTactin sepharose-packed columns and eluted in 8x 500 µl fractions. 10 µl samples were processed for SDS-PAGE followed by Coomassie Blue staining of the gel. The positions of the antigen is indicated. The molecular marker (in kDa) is shown. **(c)** Purified HBV-stC$_{AAA}$ was processed for electron microscopy. The indicated scale bar represents 200nm.

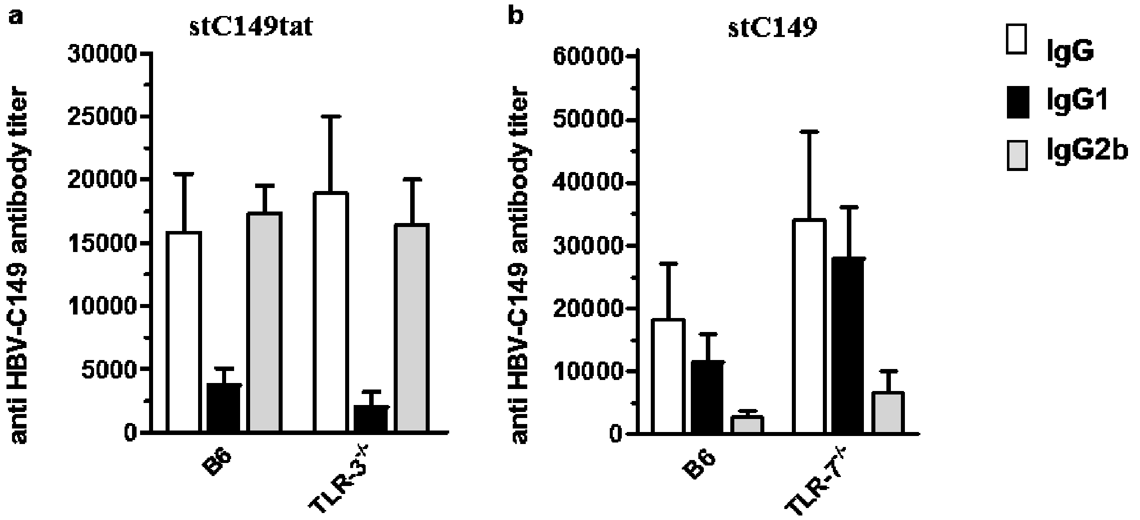

Supplementary Fig. S7

Characterization of humoral immune responses. (a) B6 and TLR3$^{-/-}$ mice were immunized with recombinant HEK-293-derived stC149tat particles (n=2/4). Three weeks post injection serum samples were analyzed for HBV core-specific IgG, IgG1 and IgG2b serum antibody titers by end-point dilution ELISA using bacterial rHBV-C149 particles as detection antigen. **(b)** B6 and TLR7$^{-/-}$ mice were immunized with recombinant stC149 produced in HEK-293 cells. Three weeks post injection serum samples were analyzed as described in a. **(a, b)** Mean specific antibody titers in sera ±□SD of a representative experiment (out of two performed experiments) are shown.

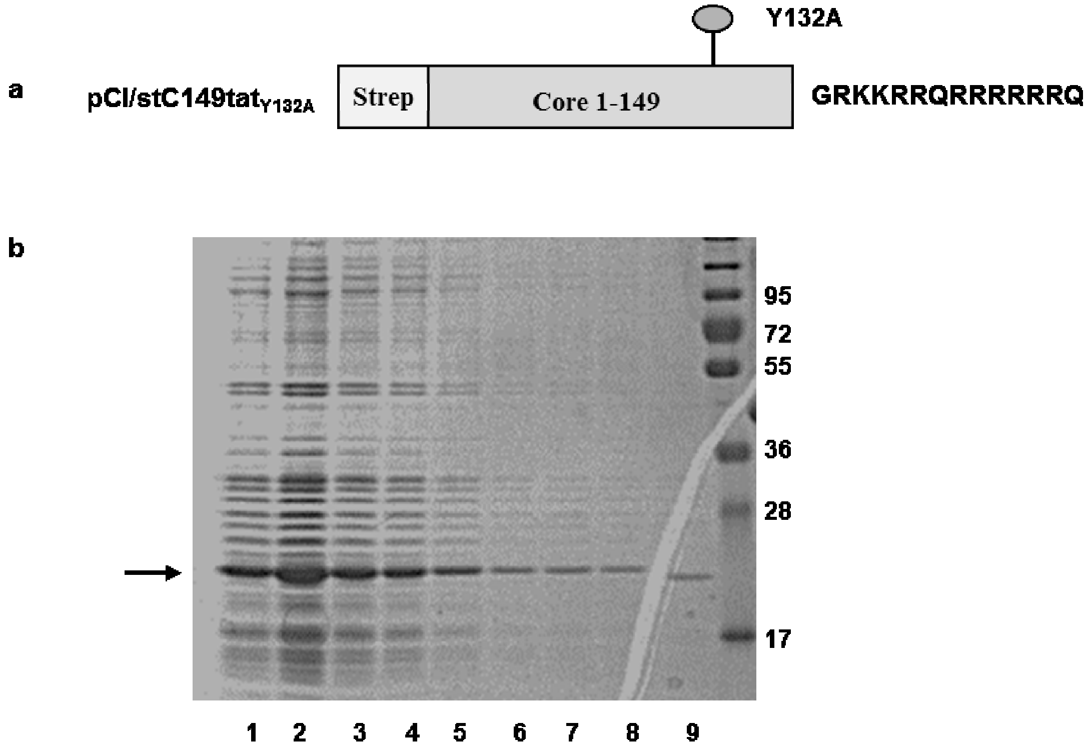

Supplementary Fig. S8

SDS-PAGE analysis of purified HEK293-derived stC149tat$_{Y132A}$. **(a)** Schematic presentation of the assembly-deficient stC149tat$_{Y132A}$ protein. The substitution of the tyrosine residue at positions 132 to alanine is indicated. **(b)** HEK-293 cells were transiently transfected with the pCI/stC149tat$_{Y132A}$ vector. The protein was purified from cell lysates using StrepTactin sepharose-packed columns and eluted in 9x 500 µl fractions. 10 µl samples were processed for SDS-PAGE followed by Coomassie Blue staining of the gel. The position of stC149tat$_{Y132A}$ is indicated (arrow) and the molecular weight marker is shown.

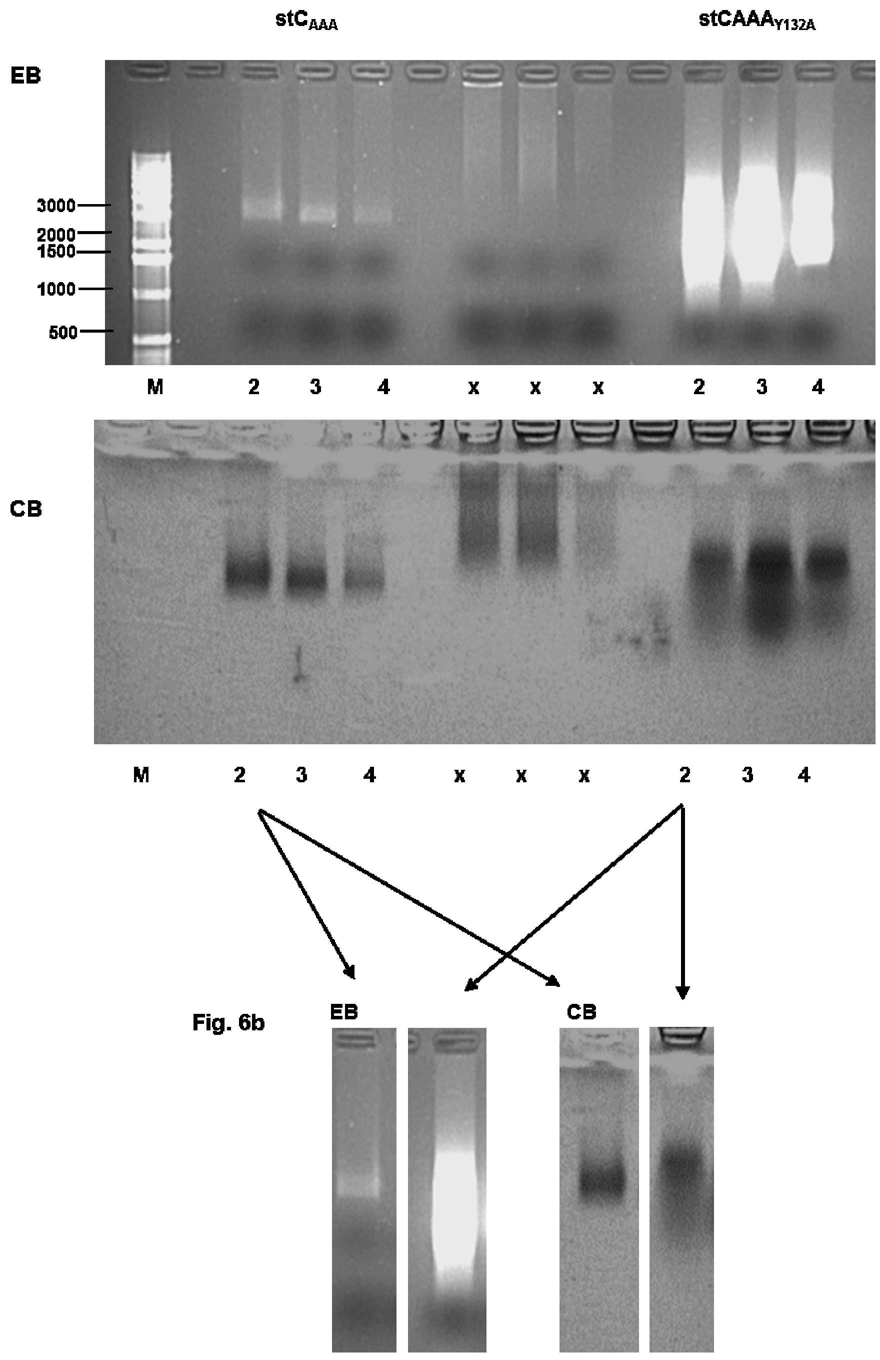

Supplementary Fig. S9

Generation of cropped Figure 6b. (a) Fractions 2 to 4 of purified HBV-stC$_{AAA}$ and stCAAA$_{Y132A}$ antigens expressed in HEK-293 cells were run on a native agarose gel followed by ethidium bromide (EB) and subsequent Coomassie Blue (CB) staining of the gel. 1kb DNA ladder is shown. Arrows indicate the origin of stC$_{AAA}$ and stCAAA$_{Y132A}$ preparations (fractions 2) used for generating Figure 6b (lower panel). x: samples not relevant for this manuscript

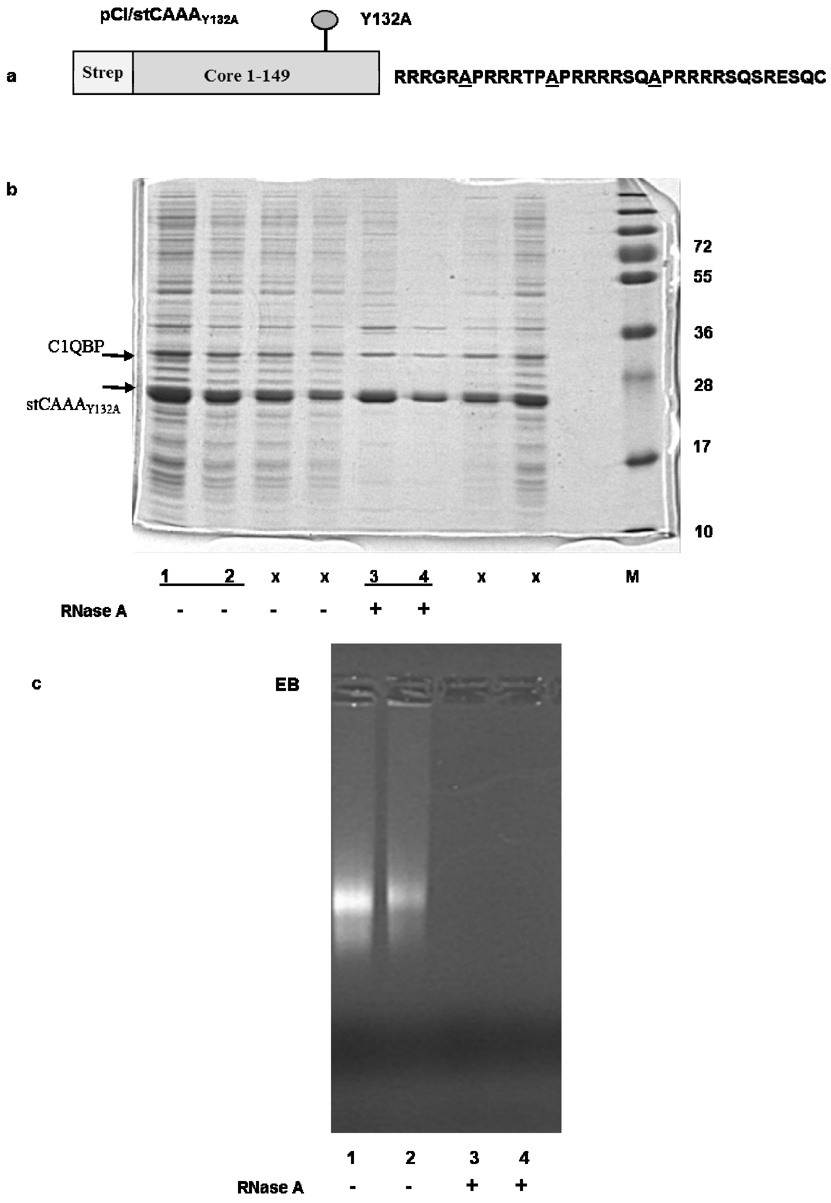

Supplementary Fig. S10

RNA-binding of HEK293-derived stC149$_{Y132A}$. (a) Schematic presentation of the non-particulate stCAAA$_{Y132A}$ protein. The substitutions of the tyrosine residue at positions 132 to alanine is indicated and the amino acid sequence of the mutant C150-183AAA domain is shown. **(b, c)** 5x10^7 (lanes 1 and 3) and 1x10^7 HEK-293 cells (lanes 2 and 4) were transiently transfected with pCI/stCAAA$_{Y132A}$. Cell lysates were lysed either with RNAse free (-; lanes 1 and 2) or RNAse A (+, lanes 3 and 4) containing lysis buffer and HBV-stCAAA$_{Y132A}$ protein was purified using StrepTactin sepharose-packed columns. **(b)** Samples of purified HBV-stCAAA$_{Y132A}$ were processed for SDS-PAGE followed by Coomassie staining of the gel. The molecular weight marker (in kDa) is shown**.** The positions of the HBV-stCAAA$_{Y132A}$ protein and the co-precipitating 32 kDa mature form (residues 74-282) of the complement component 1 Q subcomponent-binding protein (C1QBP) are indicated. x: samples not relevant for this manuscript. **(c)** Furthermore, samples of purified HBV-stCAAA$_{Y132A}$ were run on an agarose gel followed by ethidium bromide staining (EB) of the gel.

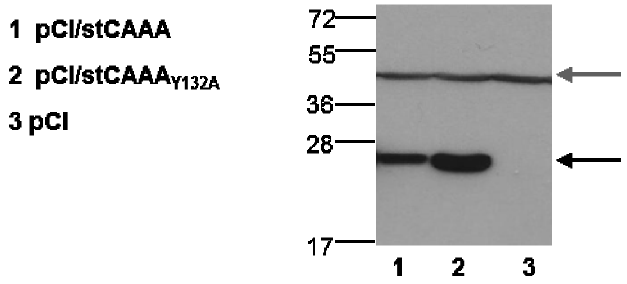

Supplementary Fig. S11

Western blot of modified HBV-stCAAA and HBV-stCAAA$_{Y132A}$. Lysates of HEK-293 cells, transiently transfected with pCI/stC$_{AAA}$ (lane 1); pCI/stCAAA$_{Y132A}$ (lane2) or control pCI (lane 3) vectors, were processed for SDS-PAGE followed by Core-specific and actin-specific western blot analysis. The positions of beta actin (grey arrow) and respective HBV core antigens (black arrow) are indicated.

Supplementary Protocols

DNA immunization with the gene gun.

Where indicated small amounts of plasmid DNA (1µg) were intradermally administered into the shaved abdominal skin using a helium driven gene gun (Helios® Gene Gun System, Bio-Rad, Hercules, CA, USA). For intradermal DNA vaccination, plasmid DNA was incubated with spermidine, loaded onto 1µm gold microcarrier particles using $CaCl_2$ (1µg DNA/0.5mg gold) and fixed in a tube (cat. no. 1652441; Bio-Rad) using polyvinylpyrrolidone (PVP). DNA-coated gold particles were injected with a helium pressure of 300 psi [1].

Construction of expression vectors.

The antigenic sequences of the different core antigens were codon-optimized and synthesized by GeneArt (Regensburg, Germany) and constructed by PCR. All constructs were cloned into the pCI vector (cat. no. E1731, Promega, Mannheim, Germany) using the *NheI* and *NotI* restriction sites. Batches of DNA were produced in *E. coli* using the Qiagen Plasmid Mega Kit (cat. no. 12183; Qiagen, Hilden, Germany). The pCI/stCAAA$_{Y132A}$ was generated by PCR using the primer 5´ccccctgccgccagaccccccc 3´, 5´gtccggatccacacgcccg 3´and the pCI/stC$_{AAA}$ as a template. The pCI/stC149tat$_{Y132A}$ vector was generated using the primers 5´ agaaggcggaggcggaggcagtaatgagcggccgcttcgagc3´ and 5´ctggcgcctcttctttcttcccacgacggttgtctcgggcag 3´ and pCI/stC$_{Y132A}$ as a template. Both constructs were cloned using the Q5® Site-Directed Mutagenesis Kit (cat. no. E0554s; NEB; Frankfurt, Germany) according to manufacturer’s instructions.

HBV core antigen expression in transfected cells

Human embryonic kidney cells (HEK-293 cells**;** ATCC® CRL-1573) were transiently transfected with the indicated DNA plasmids using the calcium phosphate method. Where indicated, cells were labelled with 100µCi ^{35}S-methionine/cysteine (cat. no. IS103, Hartmann Analytic GmbH, Braunschweig, Germany) 36h post transfection and lysed 12 h post labelling with pH 8.0 lysis buffer (100 mM NaCl, 0.5% NP40 and 100 mM Tris-hydrochloride) supplemented with Protease Inhibitor Cocktail Tablets (cat. no. 11836145001, Roche Applied Science, Penzberg, Germany). Extracts were cleared by centrifugation and precipitated with a polyclonal rabbit anti core serum and protein-A sepharose. Precipitates were processed for SDS-PAGE and subsequent fluorography of the gels. For western blot analyses, transiently transfected cells were directly lysed with a SDS-containing buffer (62,5 mM Tris-hydrochloride, 3% SDS, supplemented with 5% mercaptoethanol [pH 6,8]), 48 h post transfection and processed for SDS-PAGE and blotted on nitrocellulose membranes (cat.no. 10600006, AmershamTMProtranTM0,2µm NC, GE Healthcare, Dornstadt, Germany). Membranes were incubated with a polyclonal rabbit anti HBV core serum or mouse anti beta-actin mAb (cat.no. A2228, Sigma, Munich, Germany), followed by an incubation with HRP-labeled donkey anti-rabbit IgG (cat.no. NA934, GE Healthcare, Dornstadt, Germany) or sheep anti-mouse IgG (cat.no. NA931V, GE healthcareHealthcare; , Dornstadt, Germany). The membranes were dried and the HRP detection reagent (cat.no. WBKLS0100, Millipore, Darmstadt, Germany) was applied as recommended by the manufacturer followed by exposure to a radiography film (cat.no. 28906847, GE Healthcare, Dornstadt, Germany).

Expression of HBV-stC particles in bacteria

For prokaryotic protein expression the strep-tag/HBV-C sequence (stC) was codon optimized for bacterial expression by GeneArt (Regensburg, Germany) and cloned into the pET28a(+)

expression vector (cat.no. 69864-3; EMD Millipore, Billerica, MA, USA) using *NcoI* and *NotI*. The resulting plasmid pET28/stC was transfected into *E.coli* BL21DE3. 500 ml cultures were grown until $O.D._{600}$ reached 0.5-0.6. Thereafter, protein expression was induced by adding 1mM IPTG and the culture was continued overnight at RT. Recombinant HBV-stC particles were purified from bacterial extracts using Strep-tag purification system as described in Materials and Methods.

Transmission electron microscopy

Purified protein samples were transferred to mesh copper grids and negatively stained with 2 % uranyl acetate prior to examination on a Zeiss TEM EM10 or Joel TEM 1400 electron microscope at 100 kV.

Cytokine detection by ELISA

Spleen cells were isolated [2] and $1x10^6$ cells were stimulated *ex vivo* for two days in Ultra Culture medium with 5µg/ml of C128-139 peptide (5µg/ml). IFN-γ secretion was measured in cell culture supernatants by conventional double-sandwich ELISA using purified rat anti-mouse IFN-γ (cat.no. 551216, BD Pharmingen, Heidelberg, Germany) and biotin rat anti-mouse IFN-γ (cat.no. 554410, BD Pharmingen, Heidelberg, Germany). Extinction was analysed at 405 nm on a Spectra Max® 250 microplate-ELISA reader (Molecular Devices, Biberach, Germany) using Softmax PRO software.

References

1. Kwissa,M. et al. Efficient vaccination by intradermal or intramuscular inoculation of plasmid DNA expressing hepatitis B surface antigen under desmin promoter/enhancer control. Vaccine **18**, 2337-2344 (2000).

2. Riedl, P. *et al.* Priming Th1 immunity to viral core particles is facilitated by trace amounts of RNA bound to its arginine-rich domain. *J. Immunol* **168**, 4951-4959 (2002).
